# 

**Published:** 1894-11

**Authors:** 


### CONTENTS:

### SELECTIONS.

Article I.—Remarks on the Treatment of Exposed Pulps and Pulpless Teeth. By D. D. Atkinson, D. D. S. - 289
" II.—National Association of Dental Examiners. - - 294
" III.—Science in Dentistry. - - - - - - 299
" IV.—The Present Status of Dental Knowledge in the Medical Profession. - - - - - - 307
" V.—Homœopathic Treatment of Toothache from Pulp-capping. By Charles H. Taft, D. M. D. - - 312
" VI.—Necrosis of the Alveolar Process: How Shall it be Treated? By J. Henry Morgan, D. D. S. - - 316
" VII.—A Year's Experience with Three New Remedies. By Dr. I. N. Carr. - - - - - - - - 321
" VIII.—Tropa-cocain in Painless Extraction of Teeth. - 323
" IX.—Nitrate of Silver and its Application in Diseased Conditions of the Mouth and Teeth. By J. M. Parker. - - - - - - - - 325

### EDITORIAL, ETC.

Causes of Irregularities. - - - - - - - 328
Dental Works in the Enoch Pratt Free Library. - - - - 332
The First Quarterly Clinic of the Maryland State Dental Society. - 333

### MONTHLY SUMMARY.

Necrosis, Caused by the Non-eruption of a Deciduous Molar. - 334

Promiscuous Extraction of Teeth. - - - - - - 335

Treatment of Root Canals. - - - - - - - 335

Caries of the Alveolus. - - - - - - - 336

Aristol. - - - - - - - - - - 336

